# Validation of Glacier Topographic Acquisitions from an Airborne Single-Pass Interferometer

**DOI:** 10.3390/s19173700

**Published:** 2019-08-26

**Authors:** Delwyn Moller, Scott Hensley, Jeremie Mouginot, Joshua Willis, Xiaoqing Wu, Christopher Larsen, Eric Rignot, Ronald Muellerschoen, Ala Khazendar

**Affiliations:** 1 Remote Sensing Solutions, Barnstable, MA 02630, USA; 2 Jet Propulsion Laboratory, California Institute of Technology, Pasadena, CA 91109, USA; 3 Irvine Department of Earth System Science, University of California, Irvine, CA 92697-3100, USA; 4 Geophysical Institute, University of Alaska Fairbanks, Fairbanks, AK 99775, USA

**Keywords:** interferometry, topography, glacier

## Abstract

The airborne glacier and ice surface topography interferometer (GLISTIN-A) is a single-pass radar interferometer developed for accurate high-resolution swath mapping of dynamic ice surfaces. We present the first validation results of the operational sensor, collected in 2013 over glaciers in Alaska and followed by more exhaustive collections from Greenland in 2016 and 2017. In Alaska, overlapping flight-tracks were mosaicked to mitigate potential residual trends across-track and the resultant maps are validated with lidar. Furthermore, repeat acquisitions of Columbia Glacier collected with a three day separation indicate excellent stability and repeatability. Commencing 2016, GLISTIN-A has circumnavigated Greenland for 4 consecutive years. Due to flight hour limitations, overlapping swaths were not flown. In 2016, comparison with airborne lidar data finds that residual systematic errors exhibit evenly distributed small slopes (all less than 10 millidegrees) and nadir biases were typically less than 1 m. Similarly 2017 data exhibited up to meter-scale nadir biases and evenly distributed residual slopes with a standard deviation of ~10 millidegrees). All satisfied the science accuracy requirements of the Greenland campaigns (3 m accuracy across an 8 km swath).

## 1. Introduction

Sea level rise in many areas might be one of the largest environmental challenges of the 21st Century. Without adaptation, the cost of sea level rise could reach $14 trillion per year by 2100 [1]. Yet despite its importance, sea level rise remains one of the most poorly predicted impacts of certain activities. Most of this uncertainty stems from our inability to predict how the great ice sheets in Greenland and Antarctica, which contain ice equivalent to 65 m of sea level, will respond to human-caused global warming [2].

Dynamic changes in the marine-terminating glaciers of Greenland and Antarctica, were cited by both the 2007 and 2013 IPCC Reports as being the largest source of uncertainty in sea level rise projections [3,4] in part because existing numerical models are unable to represent them accurately enough to reliably predict their evolution. To provide the critical data needed to accurately measure glacier volume changes radar and laser altimetry, both airborne and from orbit, have become increasingly indispensable. NASA’s Airborne Topographic Mapper (ATM) has been surveying Greenland glaciers since the early 1990s [5]. More recently, radar measurements covering all of the ice sheet of Greenland were made by the CryoSat-2 mission [6]. The recently launched ICESat-2 mission promises laser altimetry measurements at high spatial resolution and temporal sampling [7]. As part of such efforts, a swath mapping radar was developed which can provide elevation maps independent of cloud cover and solar illumination. The airborne Glacier and Ice Surface Topography Interferometer (GLISTIN-A) instrument was first demonstrated in 2009 as part of NASA’s International Polar Year activities [8]. Since then the system was updated for enhanced performance and to support NASA science operations and these capabilities were successfully demonstrated in a campaign to Alaska in 2012 where glacier and sea-ice flights were conducted. The GLISTIN-A system has been incorporated for operations by NASA’s Jet Propulsion Laboratory (JPL) and is now being leveraged for a diversity of science applications including snow-topography mapping, flood-plain mapping and volcanology (e.g., [9,10]). Perhaps most notably, GLISTIN-A is flying as a critical capability for NASA’s Earth Ventures Mission, Oceans Melting Greenland (OMG) [11], circumnavigating Greenland annually to measure changes in 90% of the ice-sheets marine terminating glaciers since 2016.

In this paper we will present the first validation results of the GLISTIN-A interferometer. Since the fidelity of any calibration is inherently finite and we will validate the stability and accuracy of that calibration in the context of the science application. In the Results discussion we start Section 2.1 with an overview of the GLISTIN-A design after which Section 2.2 introduces the sources of systematic errors and how they manifest for a swath interferometer. Section 2.3 summarizes the 2012 Alaska campaign and validation results. Section 2.4 then shows validation results from the first two years of the OMG campaigns. Section 3 provides general discussion followed by Materials and Methods in Section 4 and Concluding statements in Section 5.

## 2. Results

### 2.1. The GLISTIN-A Interferometer

GLISTIN-A first demonstrated the ability to generate high resolution, high-precision height maps during NASA’s IPY activities [8] in 2009. After that, the NASA Earth Science Technology Office funded the transition of the IPY configuration to an improved, permanently-available, Ka-band UAVSAR configured interferometer. Upgrades included new Ka-band up- and down-converter chains, state-of-the-art solid state power amplifiers (SSPA) and an integrated calibration loop. These improvements have resulted in more peak transmit power and the ability to ping-pong, (i.e., both antennas are capable of transmit and receive) which improves the vertical accuracy of data by a factor of two. By the addition of these capabilities, GLISTIN-A has an improved swath coverage of greater than 10 km as it will typically fly at higher altitudes but with the same sampling geometry (look angles ranging from 15 to 45 degrees) for similar performance. Table 1 summarizes the key system parameters including the differences from the initial and upgraded system and the predicted height precision performance for a 30×30 m spatial posting at the antenna boresight look angle of *θ* = 31°. Note that the estimated height precision is estimated from the measured interferometric correlation and is dominated by the instrument random noise. This relative error is high frequency and will scale with spatial averaging of uncorrelated pixels or independent samples. Systematic errors on the other hand are correlated and can not be reduced in the same manner.

The science measurement requirement levied on the GLISTIN-A instrument at the design phase were better than 50 cm height accuracy for pixel postings of 30 m × 30 m resolutions over a swath of 8 km [8]. The instrument height-error budget comprises several sources:

1. Random errors due to thermal noise, ambiguities and multiplicative noise ratios (this is the precision quoted in Table 1 and will improve with spatial averaging).
2. Geophysical contributions from differential electromagnetic wave penetration into snow cover, volume decorrelation and snowfall and compaction. Note that for an interferometer this contribution is the resulting shift in the mean interferometric phase center.
3. Systematic errors due to limitations in the knowledge of the platform attitude, baseline and antenna thermal deformations.

With respect to item #1 above, Moller et al. [8] discusses in detail the interferometric design and calibration methodology including an assessment of the random errors (precision) prior to the system upgrades summarized in Table 1, however that analysis remains valid for GLISTIN-A.

Geophysical contributions to height error (item #2 above) are assessed in Hensley et al. [12] which analyses the impact of bias due to interferometric penetration into snow cover. In addition to a comprehensive modeling assessment, coincident lidar and GLISTIN-A height measurements were compared with surveyed ground-control points (corner reflectors) at Greenland’s Summit. They found approximately 30 cm of penetration that compared with scattering models with a surface-to-volume scattering ratio of −20 dB. It is to be expected that the penetration depth at Ka-band would be greatly reduced in comparison to lower frequencies of operation, but it is also important to note that the interferometric penetration is indicative of the location of the interferometric phase center and will be less than the $\frac{1}{e}$ electromagnetic penetration depth.

In this paper we present a quantitative assessment of the systematic errors for the operational GLISTIN-A sensor followed by validation of those assessments thereby addressing item #3 above.

### 2.2. Systematic Error Contributions

To meet the accuracy requirements, systematic error sources must either be calibrated, or the overall design must be such that they are small enough to be neglected. In addition to the interferometric penetration bias the sources of systematic errors affecting the interferometric height measurement can be summarized by:

1. The relative phase drift of the receivers, δϕ
2. Aircraft attitude knowledge, δα and position uncertainty, $\delta h_{p}$
3. Errors in the transition of the radar timing measurement to the geometric range, δR;
4. Interferometric baseline change due to thermal distortions, δB;
5. Isolation between the receivers.

The isolation in the Ka-band chain was measured to exceed 80 dB (greater than 90 dB between antennas), making this a negligible factor. The contribution to the systematic height errors due to the other sources can be expressed as [8,13]:

(1) $$\delta h=\frac{Rsin(\theta )}{2kBcos(\theta -\alpha_{B})}\delta \phi +Rsin\left(\theta \right)\delta \alpha -\frac{R}{B}tan\left(\theta -\alpha_{B}\right)sin\left(\theta \right)\;\delta B-cos\left(\theta \right)\delta R+\delta h_{p}$$

where *R* is range to the surface pixel, θ is the look angle to the surface, k=2π/λ and λ is the transmitted wavelength. *B* is the baseline distance between the two antennas and $\alpha_{B}$ is the baseline orientation angle, i.e., the angle the baseline vector (vector between the two antenna phase centers) makes with respect to the local horizontal. To first order, the geometric knowledge error (δα) and the residual differential phase errors (δϕ) manifest as a height shift and linear tilt in the cross-track dimension *x* (where x=Rsin(θ)). These can be corrected with two tie points. Baseline knowledge (δB) and range-timing errors (δR) appear as a height shift and quadratic height distortion across-track. Platform position errors ($\delta h_{p}$) manifest as a direct positional shift.

As detailed in [8], the post-calibration systematic errors are estimated as follows:

- The baseline length error after calibration is assumed to be 1/3 the *a priori* surveyed value (0.1 mm);
- Thermal variations in the baseline are bounded by design (δB<2 μm over a temperature range of −70 °C to +70 °C as detailed in [8]);
- The peak variation in the baseline orientation, α, as limited by Embedded GPS INU (EGI) measurement accuracy flown for these campaigns was assessed to be ~14 mdeg (peak to peak);
- The platform position error is based on the post-processed GPS accuracy of 5 cm; and
- Drift in the differential phase is monitored via an internal calibration loop which is coupled through the entire transmit/receive chain with the exception of the front-end switches and antennas. We have allocated 1 degree to the unknown drift.

**Figure 1:**
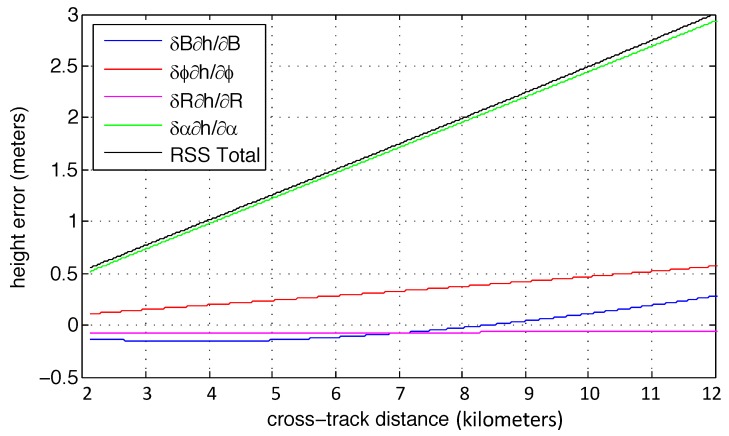
An estimate of the contributions of residual error terms to a systematic height artifact across the swath. Note that the contibuting factors can vary with sign so the slope can vary in both directions. Uncertainty in knowledge of the baseline roll, δα is the dominant source of error for GLISTIN-A using the EGI flown prior to 2018.

Figure 1 illustrates how these error sources (Equation (1)) might manifest across the swath using the assumptions listed above. Note that thermal variations in the baseline are negligible and the platform position error of ±5 cm is not included either for simplicity, but it is included in the root-sum-square total error. By far the dominant error source is knowledge of the baseline orientation.

Consistent with the system design and calibration methodology, we have observed that the post-calibration residual systematic errors manifest as a slowly time-varying linear height ramp and offset in the cross track direction. Quadratic behavior is not evident indicating those contamination sources are minimal. Small residual tilts and biases from instrument calibration and attitude accuracy knowledge can be reduced using overlapping swaths and/or tie points. In Section 2.3 overlapping swaths were mosaicked to enforce consistency and mitigate residual trends. In Section 2.4 we assess the post-calibration data collected for the NASA Ventures Oceans Melting Greenland (OMG) campaign when compared with lidar in the absence of tie points or other constraining data.

### 2.3. Alaska Glacier Acquisitions

#### 2.3.1. Campaign Summary

The engineering upgrades were completed in 2012 and GLISTIN-A successfully concluded its engineering and calibration efforts in early 2013. In April 2013, a campaign to collect glacier and sea-ice data was conducted in Alaska. Those collections demonstrated the capability for high-precision, high resolution mapping of ice surface topography from the GIII with swaths in excess of 10 km. On 24 April 2013 the NASA GIII, operated by Armstrong Flight Research Center transited to Fairbanks, AK from its base in Palmdale, CA. En route, data was collected over Columbia glacier. Just two local flights were conducted out of Eielson Air Force Base on 25 and 26 April. 25 April was dedicated to glacier mapping of primarily marine terminating glaciers including Hubbard, Yahtse-Malaspina, Bering and a number of tracks over Glacier Bay. While not marine-terminating, Nabesna was opportunistically imaged en-route from Fairbanks. 26 April was focused primarily on a sea-ice data collection in the Beaufort Sea as a proof-of-concept mission to establish the feasibility of measuring freeboard. 27 April saw the return to Palmdale, once again via Columbia glacier to collect a second observation with a 3 day separation from the first.

We focus here on the glacier collections. In each instance we collected consecutive fully overlapping swaths from opposite viewing directions. This way regions that might be shadowed in one geometry will most probably be viewed from the other. Furthermore, we were then able to mosaic the overlapping lines using the near-range of each to perform a residual tilt/bias correction for the overlapping far-range.

Based on the system implementation and calibration (as summarized in Section 2.1 and Figure 1) in addition to engineering assessments of calibrated sites and sea-ice data, we expect in the stripline (single-pass data) to have an (absolute) bias of up to 1–2 m. If ground control points are available this can be corrected. We also expect to have a slope to the data that will propagate across the swath and that both the linear (bias and slope) characteristic will be slowly time varying. In the Alaska acquisitions, successive race-tracks were acquired from opposite directions whereby, if the calibration error sources vary slowly with respect to the acquisition time frame, that is tens of minutes, the slopes across the swath would effectively cancel. Any bias term however will remain in the absence of ground-control points for absolute correction. Thereby, when the two tracks were mosaicked we enforced continuity across the swath. As will be evidenced in the validation results that follow, no noticeable slope trend is observed as a function of cross-track distance of the mosaicked GLISTIN-A swaths.

Unfortunately, due to budget limitations and aircraft logistics it was not possible to have coordinated validation flights. However over the Hubbard, and Nabesna lidar data was identified. Hubbard and Nabesna were imaged by GLISTIN-A on 25 April 2013 and by the University of Alaska Fairbanks (UAF) lidar 21 May 2013, and 19 June 2013 respectively. Columbia glacier was imaged by GLISTIN-A twice on 24 April and 27 April 2013.

**Figure 2:**
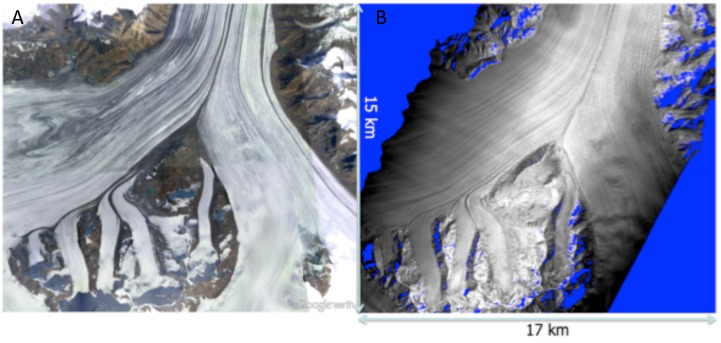
Imagery of Nabesna glacier: (**A**) Google Map image. (**B**) GLISTIN relative power image.

As an example of the GLISTIN-A imagery, Figure 2 shows an optical image from Google Earth next to a relative power mosaic generated from the two overlapping GLISTIN-A swaths posted at a horizontal resolution of 3 m.

#### 2.3.2. Validation

The University of Alaska Fairbanks (UAF) has flown lidar altimetry on glaciers in Alaska in 2013 as part of the NASA’s Operation IceBridge mission. The laser scanner was a Riegl LMS-Q240i, which has a sampling rate of 10,000 Hz and laser wavelength of 905 nm. The UAF altimetry data are comprised of a series of point measurements on the surface of glaciers recorded from an aircraft. Each point is derived from a pulsed laser range measurement combined with aircraft Global Positioning System Inertial Measurement Unit (GPS/IMU) positioning and orientation measurements. The footprint on the ground of the laser shot points is on the order of 20 cm in diameter with a vertical precision of about ±30 cm and the swath width covered by the shots is typically of 500 m. For our comparisons, the lidar shots are rasterized on the 3×3 m GLISTIN grids (UTM projection). Multiple lidar shots per grid pixel are averaged. Both datasets are converted to the same elevation datum (WGS84).

**Figure 3:**
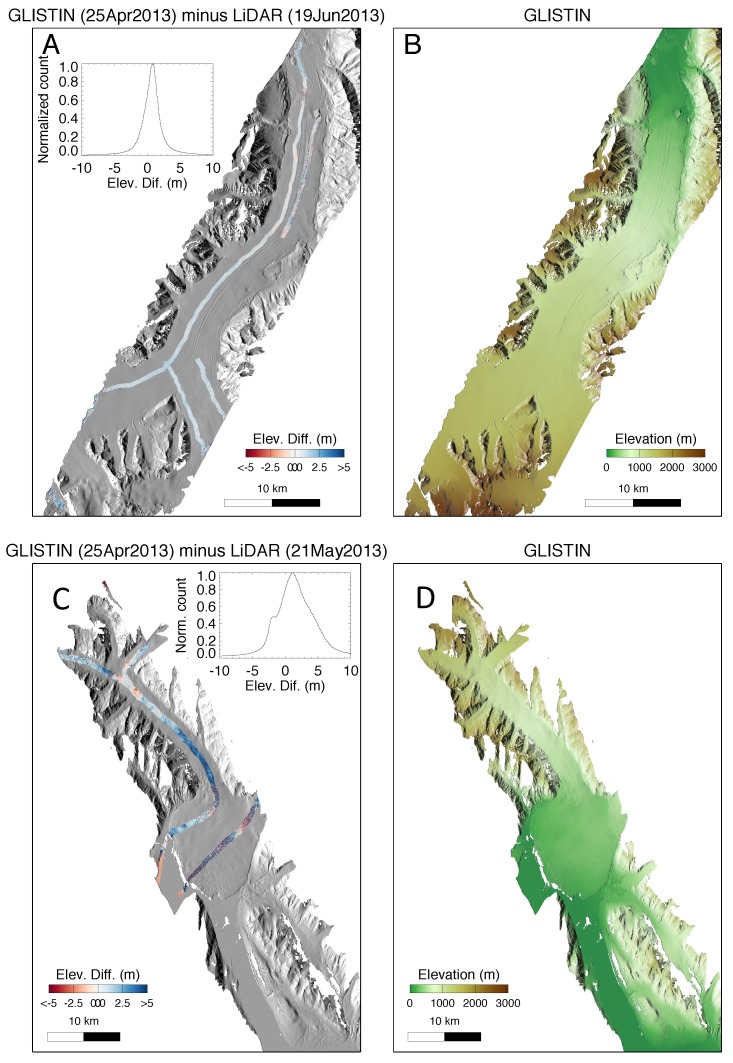
(**A**) GLISTIN-A shaded relief height map of Nabesna glacier overlaid with lidar track and GLISTIN-A—lidar elevation differences (see also inset). (**B**) GLISTIN-A digital elevation map of Nabesna generated from a mosaic of two overlapping GLISTIN-A swaths. (**C**) GLISTIN shaded relief height map of Hubbard glacier overlaid with lidar track and GLISTIN-A—lidar elevation differences (see also inset). (**D**) GLISTIN-A digital elevation map of Hubbard generated from a mosaic of two overlapping GLISTIN-A swaths.

Figure 3 shows the height map mosaics generated from the GLISTIN-A data with the lidar tracks overlaid. The lidar was flown along the center of the glacier including the both forks as seen in Figure 3. A nearly two months after the GLISTIN-A acquisition (GLISTIN-A 25 April 2013 vs. lidar 10 June 2013). The time-separation is unfortunate and a source of error in the comparison. Therefore, the differences in elevation are not only reflecting instrument accuracy but also surface elevation change and glacier displacement. Even so, Figure 3A shows the radar and the lidar compare very favorably. The mean difference is 0.8 m and the standard deviation is 1.7 m. Note that no trending is observed across the GLISTIN-A mosaic implying that if there were a slope the mosaicking process has effectively removed it, and that the mean is well within the expected range (better than 1–2 m absolute).

Similarly, the lower figures of Figure 3C,D show a comparison between GLISTIN-A and lidar over Hubbard glacier. In this instance, although the time period of separation is less (GLISTIN-A 25 April 2013 vs. lidar 21 May 2013), the observed differences are greater than those observed for Nabesna. However, examination of the height differences along the lidar tracks (Figure 3C) reveal spatially correlated “change” features varying from low-frequency differences in the Northern regions to high spatial frequency differences in the two outflow tracks resulting from crevasse movement. Importantly, the change features do not appear to have any systematic trending with respect to the radar swath, but rather are correlated with topography and reasonably attributed to topographic change. As a result the standard deviation of the differences is significantly wider: 3.7 m (compared with 1.7 m for Nabesna). The mean difference is 1.2 m, again within the expected range.

**Figure 4:**
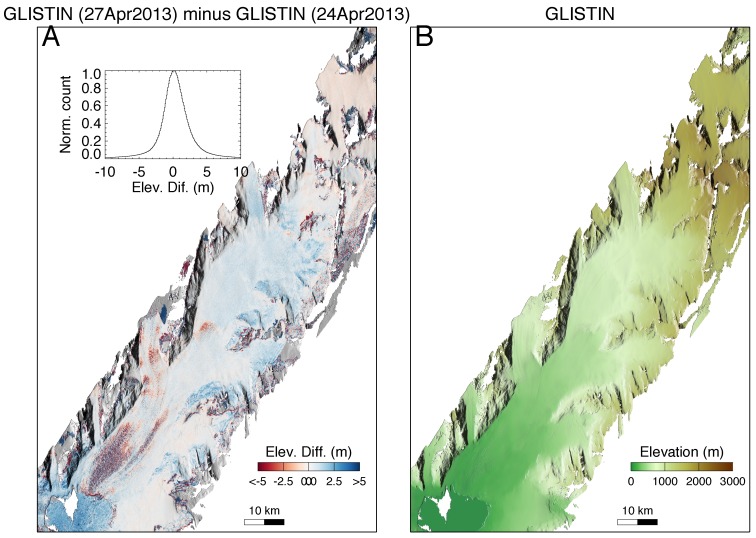
Columbia glacier: (**A**) Height difference maps observed on Columbia glacier over a 3-day interval (see also inset). (**B**) GLISTIN-A digital elevation map generated from a mosaic of two overlapping GLISTIN-A swaths collected 24 April 2013.

Finally Figure 4 shows results from the two days of GLISTIN-A acquisition over Columbia glacier. This is valuable to test the self-consistency and repeatability of the instrument. Comparison between the two height mosaics looks very good with a mean difference of ~−0.2 m. Again, no systematic trends across the swath are observed.

Heavy crevassing can be observed, especially in the lower Southwest outflow area which appears “purple” in the difference map of Figure 4A due to alternating red (negative) and blue (positive) height differences. Movement of those crevasses within the 3 day separation widens the difference distribution resulting in a standard deviation of 3.6 m. Note that the ability to track motion of features like crevasses with high spatial and temporal resolution can be exploited to derive surface velocity maps via feature tracking.

These Alaska validation results illustrate the ability to process high-accuracy DEMs without a need for external ground control points or auxiliary data such as lidar.

### 2.4. Large Scale Glacier Mapping: Oceans Melting Greenland

**Figure 5:**
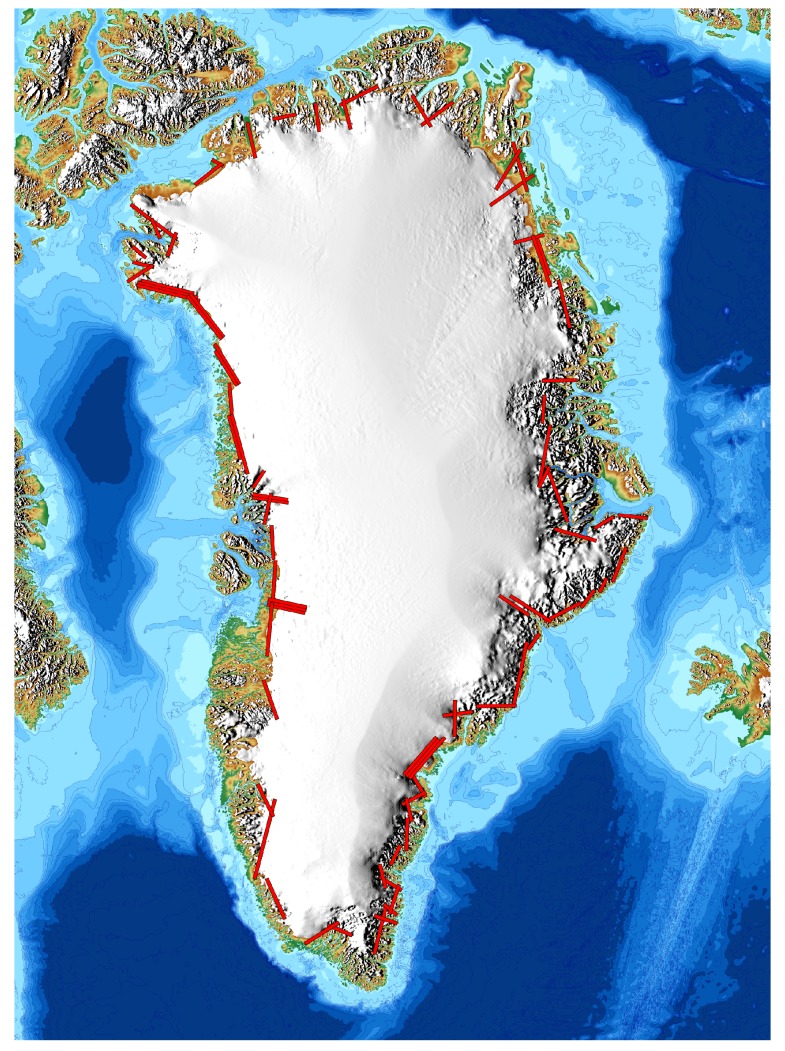
Map showing the locations of yearly GLISTIN-A data collection for OMG.

NASA’s Oceans Melting Greenland mission [11], is a five-year long airborne campaign designed to address the question: to what extent are the oceans responsible for ice loss in Greenland? In addition to an extended campaign to improve sea floor bathymetry around Greenland, OMG is conducting two sets of yearly surveys of both ocean and ice all the way around the island. See Fenty et al. [11] for a more thorough description of the bathymetry and ocean surveys. The glacier survey for OMG is carried out using the GLISTIN-A instrument and involves collection of 81 individual lines that cover more than 220 glaciers fronts all the way around Greenland (see Figure 5). To meet OMG’s science goal of quantifying ocean-ice interactions in Greenland, yearly changes in the volume of marine terminating glaciers will be computed within the last 5 to 10 km of their termini. Starting in March 2016, four such glacier surveys have now been completed, with the fourth and final survey in March 2019. OMG’s science requirements for vertical accuracy are 3 m for large-scale averages near the glacier termini. This allows fast-changing glaciers (with height changes of 10 m or more per year) to be accurately quantified on a year-to-year basis.

In 2016, the survey was cut short due to electrical issues, and only 70 of the planned glacier lines were flown over 8 flight days, leaving a gap in northwest Greenland. In 2017–2019 however, the full campaigns were achieved with 81 lines flown over a period of roughly two weeks in 2017 and equivalent extents for the latter years. This section presents validation results from 2016 and 2017.

#### 2.4.1. Validation Results

For OMG, GLISTIN-A’s role is to quantify the mean elevation changes as well as changes in glacier front positions to enable volume change estimates of over 200 marine terminating glaciers. Changes in the glacier slope are also an important dynamic quantity that will be observed annually by OMG’s GLISTIN-A campaigns. The OMG requirements of 3 m vertical accuracy at 100 m spatial resolution over an 8 km swath can be readily met by GLISTIN-A [8] but because any residual trends (tilts) are systematic and present as a slope error, they could be problematic or create confusion in interpretation.

In the Alaska flights, we planned completely overlapping swaths and used near-range data to remove any residual trends. However, this effectively doubles the flight time required and adds additional processing complexity and so was dismissed as an option for OMG due to costs and aircraft availability. Furthermore, after assessing requirements and assessing performance of existing data, the OMG science team determined that additional calibration to detrend data would not be performed as part of the OMG data delivery. Here we use airborne lidar altimetry data to validate the OMG GLISTIN-A height data and quantify the error statistics.

For validation purposes in Greenland, we identified overlapping tracks of the NASA Wallops Airborne Topographic Mapper (ATM) lidar [5]. The ATM was flying data collections for Operation IceBridge (OIB), but these flights were not coordinated with the OMG campaign. In 2016, 35 of the 70 GLISTIN-A lines had overlapping OIB ATM data.

**Figure 6:**
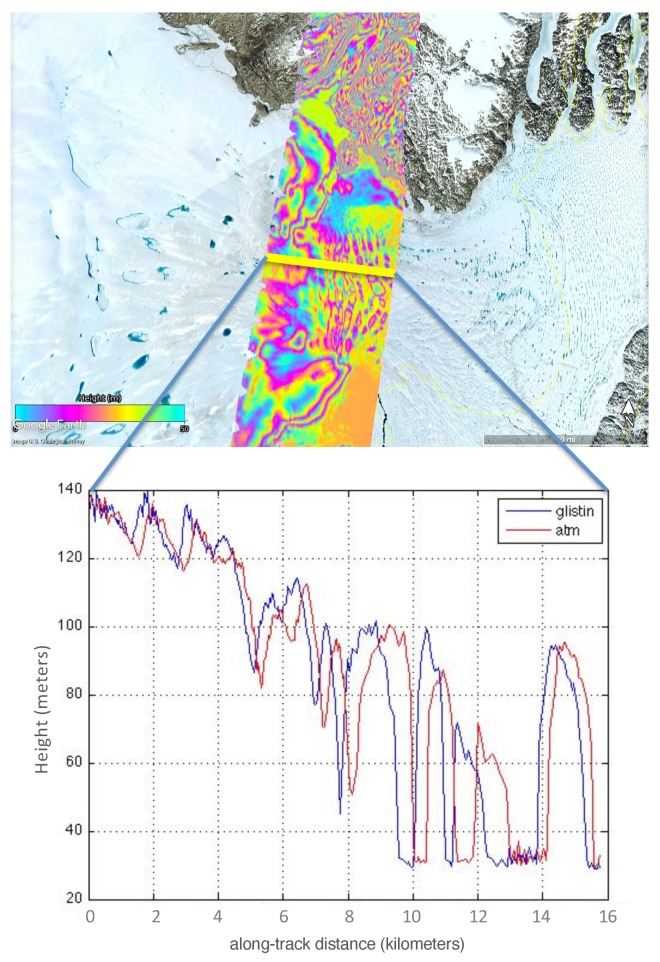
Zacharae glacier as observed by GLISTIN-A (31 March 2016) and by ATM (9 May 2016): (**Upper**) GLISTIN-A swath (colored contours, 50 m wrap) and a crossing ATM track (Yellow line) overlaid on Google Maps. (**Lower**) GLISTIN-A and ATM heights measured along the ATM track flying toward the ocean. The movement of the ice features can be clearly identified illustrating how this is an effective tool for velocity and volume change mapping but also that the dynamic glacial regions must be filtered from the validation assessment.

As an example, Figure 6 shows a comparison of an ATM line with GLISTIN-A where the ATM path flown along Zacharae glacier. ATM flew on 9 May 2016 approximately 1.5 months after the GLISTIN-A acquisition (31 March 2016). The movement of the ice in between the acquisitions can be seen quite clearly in the lower plot. This figure illustrates in two-dimensions, how using this technology for feature tracking is very apt not only for velocity mapping, but also as an accurate assessment of mass change from repeated 3-dimensional mapping capability. However, it also illustrates that we must be careful in choosing relatively static regions to compare for the height validation, such as the rocky areas surrounding the glaciers. When we do so, comparisons of ATM and GLISTIN-A result in histograms of differences similar to those observed in Section 2.3 over more static and non-crevassed regions. If relatively uniform, the impact of crevasse movement is simply to increase the variance of the comparison, still enabling an estimate of instrument systematic trends. However, as is the case here, at the lower elevations where the marine terminating glaciers approach sea-level the impact of crevasses and objects movement is not zero mean and must be excluded (as is the instance in Figure 6. We now turn our attention to identifying any residual systematic trends as a function of the cross-track distance, *x*, in the single-pass data.

As mentioned in Section 2.2 and detailed by Equation (1), residual systematic errors can manifest as a quadratic bias that propagates across the swath. Because the stability of the instrument parameters no quadratic trending of the height measurements with cross-track distance was expected nor observed, but rather any observed cross-track trend are linear (refer Figure 1).

In the comparison of Greenland single-swath (strip-map) data with ATM, because we assume the ATM to be our validating data set, the vertical height error is defined as ATM measured height—GLISTIN-A measured height. We used the ATM Level 2 IceSSN data [14]. This data is reported by resampled “platelets” each with geographic location (in latitude and longitude) and surface height (WGS84), local slope information and additional fields. The platelets are typically on the order of 30 m along-track and 80 m across track. In the case of GLISTIN-A the data is processed to a ground-projected radar-relative “sch” cartesian rectangular grid prior to regridding to geographic coordinates (latitude and longitude). The so-called “sch” coordinate system [15] is a locally defined cartesian coordinate system where “s” is along the ground track of the aircraft/radar, “c” is across-track from the aircraft/radar track (and equivalent to the cross-track distance, *x*) and “h” is the measured height relative to WGS84. The GLISTIN-A data is posted at 3 m spatial resolution (both the regridded geographic and sch products) and one can use geometric relationships to transform between the two.

In order to use ATM to validate GLISTIN-A’s performance in the cross-track direction the following process was applied for all 2016 and 2017 Greenland flight lines:

1. For a given GLISTIN-A flight line identify all ATM platelets that fall within the swath;
2. For each overlapping platelet location find the nearest GLISTIN-A 3 m pixel in sch coordinates;
3. Average a 30 m × 30 m region centered about the platelet location;
4. Calculate the height error as ATM height—GLISTIN height. Record the estimated height error’s location in latitude and longitude in addition to along-track and cross-track (radar relative/ground-projected);
5. Using geographic location, remove from comparison height differences over dynamic areas;
6. perform a least-squares fit to the remaining height errors as a function of *x* (equivalently *c*).

**Figure 7:**
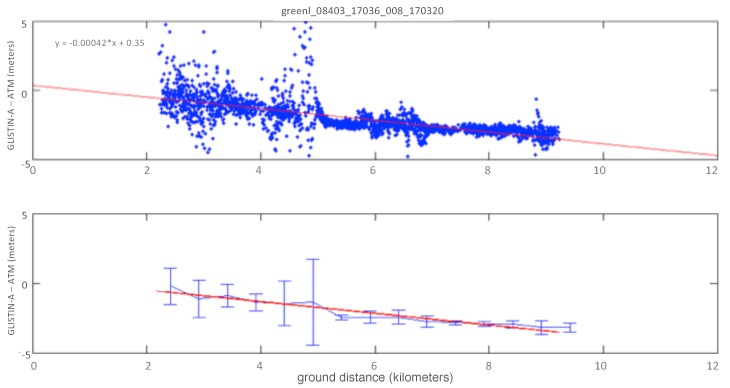
**Upper plot**: An example least-squares linear fit of GLISTIN-A to ATM data as a function of the cross-track distance. **Lower plot**: The mean and standard deviation of the height difference data compared with the linear fit above. The height difference data is binned into 500 m bins across track and the statistics for each bin is calculated. The binned means follow the linear trend well. The standard deviation is an indication of both spatial variability/dynamics and the instrument precision. For x<5000 m it is apparent that there is an influence of surface dynamics, but the fine precision further out indicates a static surface and excellent instrument precision.

In this manner we calculated the mean offsets and cross-track trends of the height errors as a function of the cross-track distance using a simple linear least-squares fit (bias and cross-track slope error). As an example the upper panel of Figure 7 shows a least-squares linear fit of the height differences (ATM-GLISTIN-A) as a function of the GLISTIN-A cross-track distance. The lower panel of Figure 7 shows the mean and standard deviation (estimated precision) of the height differences in 500 m cross-track bins, relative to the least-squares fit of the upper figure.

While, even after spatially filtering, there remains some deviations and spurious points due to surface changes one can see a consistent trend across the swath. It is also evident that any quadratic factors are negligible and a linear characterization is sufficient. The lower figure indicates the precision varies across the swath and clearly the increased spread in the near range is driven by greater topographic surface differences in this region. What is encouraging is the <0.5 m precision achieved in the 7500–8500 m cross-track distance approaching expectations for system-level random errors. This process was replicated for all 2016 and 2017 lines summarized subsequently and itemized explictly by line identifier in the tables in Appendix A.

**Figure 8:**
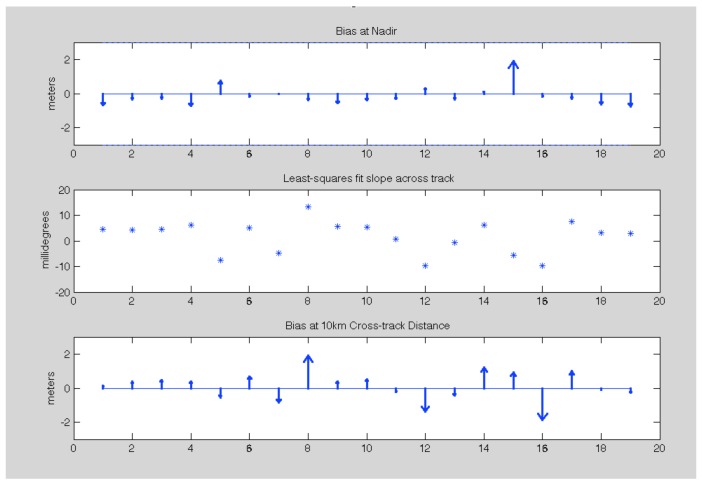
Validation results for the 2016 GLISTIN-A acquisitions. The upper plot shows the mean bias of the GLISTIN-A data when compared with ATM projected to nadir. The middle plot shows the corresponding least squares cross-track slope in millidegrees. The final plot shows the mean height bias of the GLISTIN-A data at 10 km cross-track distance (8 km swath). The dashed lines in the upper and lower plots correspond to the OMG accuracy requirements. Table A1 itemizes this information with cross reference to the flight line identifiers (in the same order as this plot).

Figure 8 summarizes the 2016 validation results applying the process detailed above over relatively static regions for the 19 lines that have validating data. The upper plot shows the magnitude of the bias for each radar data-take projected to nadir. The magnitude of the bias ranges from 1.9 m to just 1 cm and is always well below the 3 m OMG requirement (dashed lines). The middle plot shows the corresponding residual slope $\gamma =tan^{-1}\left(\delta h/dx\right)$ where δh is the vertical height error and dx is the change in cross-track distance. The slopes are generally small and ~zero-mean (−1.6±6.3 millidegrees). The largest estimated slope is 13.2 millidegrees which is equivalent to just 0.23 mm/meter slope. The third plot shows the systematic height errors when projected to 10 km cross-track distance (approximately at the 8 km swath point for comparison with OMG’s requirements). Again, the requirements for OMG are met at the outer swath for all cases assessed.

**Figure 9:**
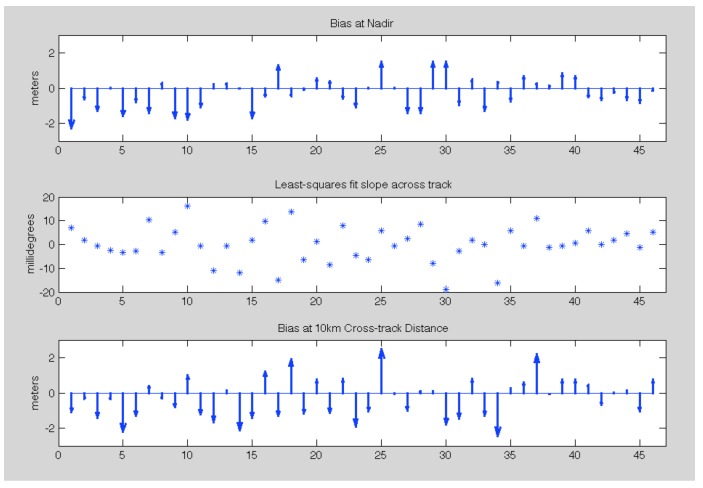
Validation results for the 2017 GLISTIN-A acquisitions. The upper plot shows the mean bias of the GLISTIN-A data when compared with ATM projected to nadir. The middle plot shows the corresponding least squares cross-track slope in millidegrees. The final plot shows the mean height bias of the GLISTIN-A data at 10 km cross-track distance (8 km swath). The dashed lines in the upper and lower plots correspond to the OMG accuracy requirements. Table A2 itemizes this information with cross reference to the flight line identifiers (in the same order as this plot).

Similarly Figure 9 shows the results for the 2017 data. Since both the ATM and GLISTIN-A flew more extensively in 2017, we were able to find a lot more lines for comparison, 46 in total. The upper plot shows biases at nadir that are still bounded within the 3 m requirement and consistent with 2016. The biases are −0.32±0.95 m. The middle plot reveals residual slopes of 0.1±7.6 millidegrees. The bottom plot shows the height errors, When propagated to 10 km cross-track distance (~8 km swath), and in all instances the 3 m accuracy requirement is easily met.

We conclude that the GLISTIN-A data meets the OMG science requirements. For OMG, these data will be used to compute volume changes near the glacier termini that are driven by speed-up or slow-down of the glaciers, in other words, dynamic volume changes. Depending on the questions and process dynamics and scales of interest, users should be aware that residual cross track trends could remain, with biases on the order of 1–3 m in the far-field.

#### 2.4.2. Estimating Yearly Volume Change

The primary science objective for OMG is to relate ocean changes to ice changes around perimeter of Greenland. For glaciers that reach the ocean and interact with it, ocean driven changes are expected to manifest as thinning or thickening that is concentrated near the glacier front [6,16,17]. To estimate these changes, swath data from subsequent years will be differenced and averaged over dynamically changing areas. Since the trend-like biases described in the previous section will not be reduced with area averaging, it is important to quantify the impact of such tilt-biases in year to year comparisons. To quantify this, we compare glacier height change signals for two typical glaciers, relative to biases estimated by comparison with co-located ATM observations.

**Figure 10:**
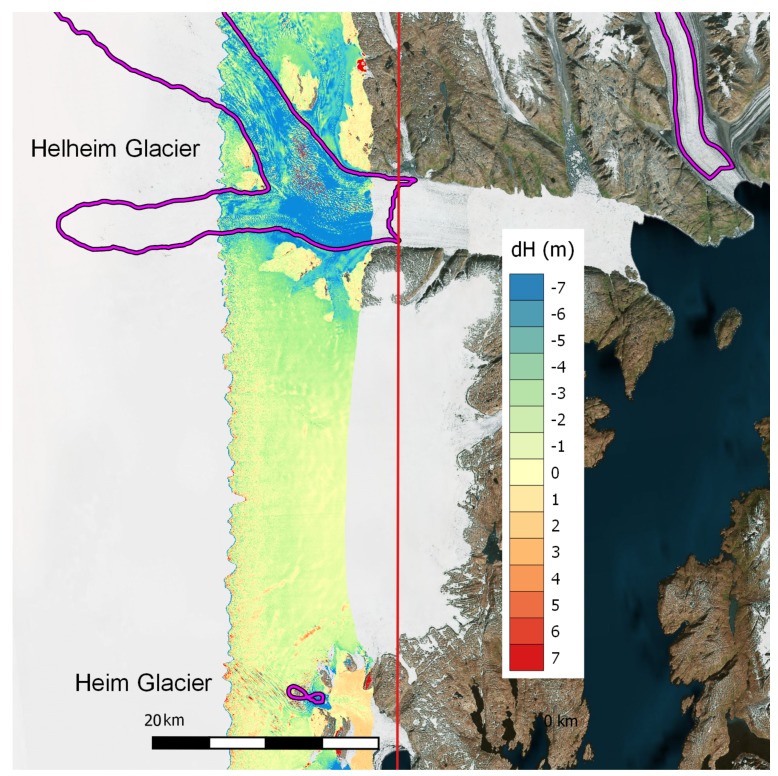
Map showing the difference (2017–2016) between GLISTIN-A elevation maps collected in subsequent years. Purple contours show regions with glacier speeds greater than 1000 m per year. The red line shows the path of the aircraft and instrument. Overlaid on Bing Aerial image of the region.

We consider line greenl_00713 in southeast Greenland. This swath covers the terminus of Helheim, one of the largest marine-terminating glaciers in Greenland. The line, which is oriented nearly north-south, also crosses the terminus of the much smaller Heim glacier to the south. The change in elevation between 2016 and 2017 is shown in Figure 10, along with contours that show glacier speeds of 1000 m per year, based on the MEaSUREs Greenland Ice Sheet Velocity from 2016 and 2017 [18]. Note that the regions with the highest speeds are also those with the most dramatic thinning.

**Figure 11:**
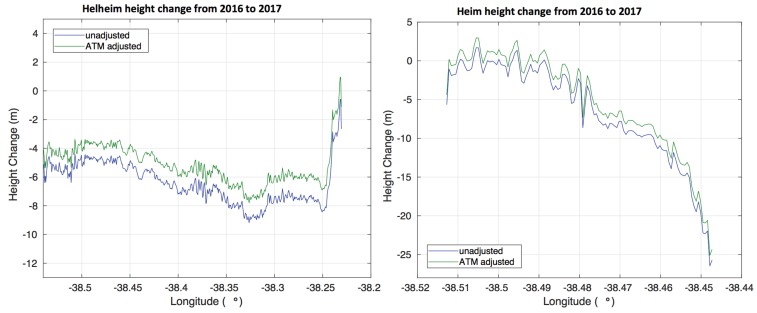
Height change as a function of longitude for Helheim (**left**) and Heim (**right**) glaciers without correction (blue) and with correction by comparison with ATM data (green). Averages are computed only over regions with velocities greater than 1000 m per year as illustrated in Figure 10.

For OMG, the primary science goal is to identify dynamic changes related to the speed up and slow down of the glaciers. It is therefore important understand the relative size of such dynamic changes, as compared to elevations changes that appear due to surface mass balance, or residual absolute bias errors in the GLISTIN-A swath data. Figure 11 shows average height change as a function of longitude for the Helheim glacier, and Heim glaciers. Two different estimates of height change are shown. The first uses GLISTIN-A data as is. For the second estimate, trend and offset biases computed in Table A1 and Table A2 were used to adjust the GLISTIN-A data to be consistent with the ATM observations. By comparing the glacier change estimates with, and without such “ATM-adjustment” it is possible to assess the impact of trends and biases of the magnitude shown in Table A1 and Table A2 on an estimate of glacier change.

From Figure 11, it is clear that dynamically-driven changes in elevation near glacier fronts are easily observed in the GLISTIN-A data regardless of whether or not they are “adjusted”. The area-averaged decrease of Helheim glacier in the region of interest was 6.42 m between 2016 and 2017, as computed using the original, unadjusted swaths. With the swaths adjusted to zero out the ATM-estimated trend and offset bias, the decrease was −5.18 m between 2016 and 2017. This ~1 m difference between the adjusted, and un-adjusted estimates of area-averaged change in Helheim is consistent with the remaining systematic bias in the swath data, as estimated in Table A1 and Table A2. If one can assume year-to-year surface cover conditions to be similar then bias due to interferometric penetration will largely cancel leaving the remaining difference dominated by systematic calibration uncertainty (per Figure 1). For Helheim, it means that area-averaged height changes, which should be closely related to volume changes near the glacier termini can be estimated with 20 to 25% accuracy each year, and larger signals should be even easier to observe.

## 3. Discussion

This paper has presented validation results and systematic accuracy assessments of the GLISTIN-A system. When concurrent overlapping swaths with opposite viewing directions were utilized as in Alaska, no significant cross-track trends were observed since self-consistency and detrending was enforced during the mosaicking process and generated without tie-points or supporting data from other instrumentation such as lidar.

For OMG, the luxury of fully overlapping swaths was not financially or logistically feasible. In the absence of ground-control points, an overall bias up to 1–2 m (absolute) and slight trending may remain. Despite these residual systematic trends the 2016 and 2017 validations easily met the OMG requirements. Even without externally-derived correction, dynamic interannual glacier volume changes are readily quantified with reasonable errors. The calibration assessment and validation will be repeated for latter years of OMG, but it is expected that the 2018 calibration will be superior due to the integration of a better EGI unit.

The assessment in this paper is relevant, not only to the performance of GLISTIN-A over land-ice but also for other science applications where exacting topographic accuracy and slopes are required. The GLISTIN-A system is now being explored in a proof-of-concept fashion for snow-depth mapping [9] and flood-plain mapping [10]. In the case of snow-depth, the requirement is for decimetric accuracy of the depth product (generated from the difference between snow-covered and snow-free observations). Not only is the accuracy requirement decimetric (albeit at coarsened spatial resolution), as opposed to meter-scale for the glaciers, but it is levied on the difference product. Thereby utilizing ground-control-points and/or static targets in addition careful flight planning for overlapping near/far swath placement and crossing tracks is critical. Similarly, flood-plain mapping requires better than 0.5 m relative accuracy at a 30 m horizontal posting. Acquisitions for flood-plain mapping in the Red River of the North Basin employed near-to-far swath overlaps and an iterative mosaicking methodology for which a large-scale self consistent digital surface model was generated via careful inter-swath detrending and validated with lidar [19].

GLISTIN-A’s swath mapping capability is not something available from ICESAT-2, CryoSat 2 or AltiKa (for example). In comparison, airborne mappers are limited to cloud-free operations with limited coverage ( e.g., 400 m vs. > 10 km for GLISTIN-A). GLISTIN-A’s capabilities (and possible future satellites) offer the possibility to do broad mapping of changes, track motion and reaches a performance level (at least in precision) similar to lasers. Systematic calibration knowledge uncertainty can be effectively mitigated for area mapping by strategic flight orientation and swath overlaps, such as demonstrated in Alaska, and obviating the need for ground-control or external data.

## 4. Materials and Methods

The methods used for validating and characterizing the performance of the GLISTIN-A system are the subject of this paper, and so largely described within the main text. Access to the data is summarized below with some supplementary details on methodology as relevant.

### 4.1. Alaska 2013

#### 4.1.1. GLISTIN-A Data

The GLISTIN-A 2013 Alaska data was collected at the conclusion of a Principal Investigator lead engineering development to demonstrate success of the system upgrades in meeting measurement requirements over glaciers and ice. As the system was not yet “operational” and the data calibration was still at the development and assessment stage, that data was not staged on the UAVSAR website (unlike the OMG and all contempory GLISTIN-A acquisitions). However, the Alaska data presented in this paper can be accessed on request to the corresponding author.

#### 4.1.2. UAF Lidar Data

The UAF lidar data is available at the NSIDC (https://nsidc.org/data/ILAKS1B/versions/1#). Reference to the data is [20]. The specific files are:

- ILAKS1B_2013_141_Hubbard.las; and
- ILAKS1B_2013_170_Nabesna.las.

### 4.2. OMG Data

#### GLISTIN-A Data

The GLISTIN-A OMG data is readily available on the UAVSAR data archive https://uavsar.jpl.nasa.gov. The data base is searchable by year, flight line identifier and/or location. The tables in Appendix A itemize each individual line identifier for which ATM data was used to compare with GLISTIN-A. For comparison with ATM, first all the ATM L2 data were pre-sorted to find all that overlapped the swath coverage of the GLISTIN-A lines. All overlapping “platelets” were saved in files with a name indicating which GLISTIN-A line(s) the data overlapped. In order to assess any trends in the cross-track relative to the flight direction, GLISTIN-A height files in “sch” coordinates were used, that is, those with a suffix “hgt.sch”. The sch coordinate system is a “radar-relative” cartesian coordinate system where the coordinates are defined relative to a linear flight track approximation (where “s” is along-track, “c” is cross-track, and “h” is height relative to the WGS84 ellipsoid). A comprehensive description of the sch coordinate system can be found in [15].

After identifying all the areas of overlap the spatial topography of the each line was assessed individually identifying the glaciers of interest and regions which were likely to have significant movement (e.g., the glacial outflow regions and sea-ice). Due to the time separation of acquisitions these regions were excluded using a spatial filtering criteria. Comparison with ATM and/or spatial imagery would typically confirm need to exclude those regions (as illustrated in Figure 6). The specific filtering can be reconstructed if the initial overlap sorting to filter the ATM data is replicated. This code (MATLAB) and line-based criteria can be furnished upon request.

### 4.3. ATM Operation Icebridge Data

The ATM data is available at the NSIDC. Reference to the data is [14]. In the case of this comparison the only fields used were location (latitude, longitude) and height. For locations (lat/lon) that overlapped a GLISTIN-A swath we simply identified the 10×10 window of 3 m pixels centered around the ATM platelet mid-point and averaged these pixels (i.e., a 30 m × 30 m average). The L2 ATM slope fields were not used although they could be applied to refine the assessment to a planar comparison. However once the areas that were obviously dominated by surface movement the error bars across the swath were assessed for every line (as per Figure 7) and found to be meter level and below and able to reveal the trends with confidence. Two independent assessments using different methodologies as noted in this paper concurred with the results within the error bounds. For these reasons we determined this 1d comparison was sufficient, especially given the time-lag between acquisitions.

## 5. Conclusions

This paper has presented the first validation results of the operational GLISTIN-A mm-wave single-pass interferometric SAR over glaciers in Alaska and Greenland.

First results used lidar data collected by the University of Alaska, Fairbanks for two Alaska glaciers: Nabesna and Hubbard. Despite several months of separation between the GLISTIN-A and the lidar acquisitions, the data sets compared very favorably. In these instances, mosaics of the radar data had been generated from consecutive fully overlapping swaths with overlapping viewing geometry. In doing so continuity is enforced and residual tilts effectively cancel. Differences observed between the radar-derived topography and the lidar measurements were found to agree well with no residual trends observed for both Hubbard and Nabesna glaciers. Furthermore comparison of mosaics collected with a 3 day separation, indicated excellent system stability with a negligible (~20 cm) mean difference between the two, with crevasse movement dominating the variance.

In the case of the Ocean’s Melting Greenland mission, the luxury of fully overlapping opposite-viewing swaths was not an option due to flight-time limitations. For these datapoints, we have assessed and quantified where possible residual systematic biases. Our validation assessment used ATM lidar data collected as part of Operation Ice-Bridge. The time of separation between acquisitions was generally weeks to months and thus areas of comparison were spatially filtered for relatively static regions, generally away from the lower outflow areas. Our assessment of 2016 and 2017 OMG data confirmed our prediction that any residual systematic trends would manifest as a small linear characteristic (as quadratic error terms were predicted by design and measurement to be negligible) propagating across the swath. Resultant bias and slope terms were found to lie within requirements for OMG, but for more exacting science requirements the calibration could be further improved by applying tie points where such ancillary data is available.

Under OMG, GLISTIN-A data for over 90 per-cent of Greenland’s marine terminating glaciers has been collected for four successive years and will now be used to assess interannual ice volume-changes of those glaciers.

## Figures and Tables

**Table 1:** Summary of Upgrades and Mapping Performance.

System Parameters		
Center Frequency (GHz)	35.66	
Bandwidth (MHz)	80	
Polarization	HH	
Look angle range (deg)	15–50	
Single look slant-range resolution (m)	1.8	
Single look along-track resolution (m)	0.25	
Baseline length (m)	0.25	
Baseline angle (deg)	45	
	**IPY**	**GLISTIN-A**
Peak Transmit Power (W)	40	56
Receive Losses (dB)	5	2
Ping-pong	no	yes
Height precision for 30 × 30 m posting and 31 degree boresite (cm)	14	17
Nominal Flight Altitude (km)	7	12
Nominal Swath	6	11

## Appendix A

**Table A1:** Summary of 2016 lines validated with ATM data summarizing residual linear trends (GLISTIN-ATM).

Flight Line Identifier	Height Bias (m)	Slope (mm/m)	Slope (mdeg)
greenl_00713_16031_000_160323	−0.67	0.08	4.6
greenl_00806_16043_002_160331	−0.35	0.08	4.4
greenl_02206_16035_001_160326	−0.30	0.08	4.4
greenl_02705_16035_000_160326	−0.73	0.11	6.3
greenl_04582_16033_001_160324	0.75	−0.13	−7.5
greenl_05600_16027_002_160321	−0.19	0.09	5.0
greenl_06602_16028_005_160322	−0.01	−0.08	−4.7
greenl_07705_16043_001_160331	−0.39	0.23	13.2
greenl_09302_16026_001_160320	−0.57	0.10	5.6
greenl_09803_16026_007_160320	−0.40	0.09	5.3
greenl_09804_16026_009_160320	−0.32	−0.01	0.6
greenl_09807_16031_001_160323	0.32	−0.17	−9.7
greenl_13507_16031_004_160323	−0.33	−0.01	−0.7
greenl_15601_16027_009_160321	0.12	0.11	6.3
greenl_18016_16026_010_160320	1.90	−0.10	−5.7
greenl_21401_16037_016_160330	−0.17	−0.17	−9.7
greenl_23401_16043_003_160331	−0.30	0.13	7.5
greenl_27202_16026_002_160320	−0.65	−0.06	3.2
greenl_27202_16026_003_160320	−0.77	0.05	2.9

**Table A2:** Summary of 2017 lines validated with ATM data summarizing residual linear trends (GLISTIN−ATM).

Flight Line Identifier	Height Bias (m)	Slope (mm/m)	Slope (mdeg)
greenl_00713_17029_000_170313	−2.3	0.12	6.9
greenl_00806_17037_001_170321	−0.63	0.03	2.0
greenl_02705_17033_000_170315	−1.3	−0.06	−3.3
greenl_04582_17028_008_170311	0.04	−0.04	−2.4
greenl_05601_17031_000_170314	−1.60	−0.06	−3.6
greenl_06603_17028_005_170311	−0.79	−0.05	−2.8
greenl_07705_17037_000_170321	−1.40	0.18	10.3
greenl_08009_17036_011_170320	0.29	−0.06	−3.3
greenl_08907_17031_013_170314	−1.70	0.09	5.3
greenl_09303_17027_094_170311	−1.80	0.28	16.0
greenl_09800_17034_003_170317	−1.10	−0.01	−0.6
greenl_09807_17029_001_170313	0.23	−0.19	−10.9
greenl_11309_17028_001_170311	0.26	−0.01	−0.7
greenl_13507_17029_004_170313	−0.02	−0.21	−12.0
greenl_14921_17034_008_170317	−1.70	0.03	1.5
greenl_15207_17038_002_170322	−0.48	0.17	9.7
greenl_15603_17034_007_170317	1.30	−0.26	−14.9
greenl_17104_17027_097_170311	−0.49	0.24	13.8
greenl_18017_17034_006_170317	−0.09	−0.11	−6.3
greenl_20604_17035_001_170317	0.56	0.02	1.1
greenl_21401_17036_012_170320	0.38	−0.15	−8.6
greenl_23401_17037_002_170321	−0.59	0.14	8.0
greenl_31505_17029_003_170313	−1.10	−0.08	−4.4
grland_22569_17028_007_170311	0.04	−0.11	−6.3
greenl_00605_17028_004_170311	1.50	0.10	5.5
greenl_00806_17037_003_170321	0.02	−0.01	−0.8
greenl_01508_17034_001_170317	−1.40	0.04	2.4
greenl_01508_17035_000_170317	−1.40	0.15	8.6
greenl_02104_17031_004_170314	1.50	−0.14	−8.0
greenl_02206_17033_001_170315	1.50	−0.33	−18.9
greenl_02901_17031_005_170314	−0.95	−0.05	−2.7
greenl_04584_17029_012_170313	0.51	0.03	1.7
greenl_04802_17029_002_170313	−1.3	0.00	−0.3
greenl_08403_17036_008_170320	0.36	−0.28	−16.0
greenl_10301_17037_010_170321	−0.74	0.10	5.7
greenl_16611_17038_000_170322	0.69	−0.01	−0.3
greenl_17909_17034_002_170317	0.28	0.19	10.9
greenl_17914_17037_011_170321	0.13	−0.02	−1.3
greenl_21203_17038_004_170322	0.86	−0.01	−0.5
greenl_21203_17038_005_170322	0.67	0.01	0.5
greenl_24901_17037_004_170321	−0.52	0.10	5.7
greenl_26402_17035_020_170317	−0.67	0.00	−0.1
greenl_27204_17027_095_170311	−0.27	0.03	1.6
greenl_31310_17036_009_170320	−0.66	0.08	4.8
greenl_33405_17031_012_170314	−0.83	−0.02	−1.0
greenl_34201_17037_006_170321	−0.16	0.09	4.9
